# Computational screening and functional tuning of chemically stable metal organic frameworks for I_2_/CH_3_I capture in humid environments

**DOI:** 10.1016/j.isci.2024.109096

**Published:** 2024-02-06

**Authors:** Haoyi Tan, Guangcun Shan

**Affiliations:** 1 School of Instrumentation Science and Opto-electronics Engineering, Beihang University, Beijing 100083, China; 2 Department of Materials Science and Engineering, City University of Hong Kong, Hong Kong SAR, China

**Keywords:** Natural sciences, Chemistry, Chemical engineering

## Abstract

High chemical stability is of vital significance in rendering metal organic frameworks (MOFs) as promising adsorbents for capturing leaked radioactive nuclides, under real nuclear industrial conditions with high humidity. In this work, grand canonical Monte Carlo (GCMC) and density functional theory (DFT) methods have been employed to systematically evaluate I_2_/CH_3_I capture performances of 21 experimentally confirmed chemically stable MOFs in humid environments. Favorable structural factors and the influence of hydrophilicity for iodine capture were unveiled. Subsequently, the top-performing MIL-53-Al with flexible tunability was functionalized with different functional groups to achieve the better adsorption performance. It has been revealed that the adsorption affinity and pore volume were two major factors altered by the functionalization of polar functional groups, which collectively influenced the iodine adsorption properties. In general, this work has screened the chemically stable high-performance MOF iodine adsorbents and provided comprehensive insights into the key factors affecting I_2_/CH_3_I uptake and separation in humid environments.

## Highlights

• Chemically stable MOFs are screened for I2/CH3I capture in humid environments
• Structure-property relationships of MOFs are revealed via GCMC and DFT
• Surface functionality of MOFs is performed to enhance the adsorption performance

## Graphical abstract

**Figure:**
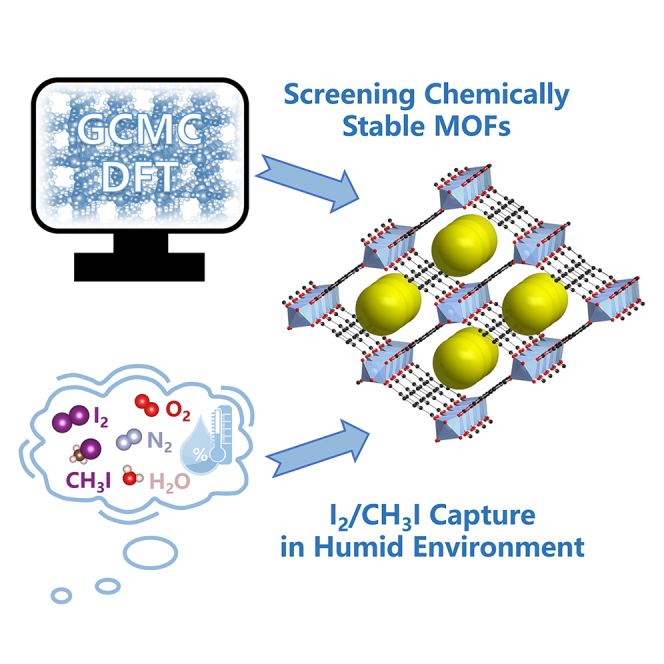

## Introduction

In contrast to traditional fossil fuels, nuclear energy is a promising green energy source to power the global economy, due to its emission-free and high energy density properties.^1^^,^^2^ However, the production of nuclear power comes with safety concerns.^3^ For instance, the leakage of volatile radioactive iodine nuclides (I^129^ and I^131^) in the nuclear accidents or during the used nuclear fuel reprocessing, poses serious threats to both the environmental safety and human metabolic health.^4^ Consequently, there is a pressing need to develop efficient methods to achieve iodine nuclides capture and separation for ensuring nuclear safety.^5^^,^^6^ The existing methods for volatile iodine nuclides removal include the precipitation, dry dedusting, wet scrubbing, adsorption, etc.^7^ Among these methods, adsorption techniques using porous adsorbents have proved the superiority, which is attributed to their high removal efficiency, simple operation and design process, good system reliability and low maintenance costs.^8^ Various traditional porous materials have been tried and used for iodine adsorption, typically including the activated carbon and silver-exchanged zeolites (AgZ).^9^^,^^10^ However, the former exhibits the low separation efficiency and poor high-temperature resistance; while the latter have the drawbacks of low adsorption capacities and adverse impact on environment. All of these problems limited their practical applications.^5^

As a novel porous crystalline material, metal-organic frameworks (MOFs), formed through self-assembly coordination polymerization of metal ions and organic ligands, possess exceptional structural characteristics—high porosity, large specific surface area and pore volume, wide range of pore size, and good tunability for surface functionalization.^11^^,^^12^^,^^13^ The aforementioned attributes render the MOFs as promising candidates for iodine adsorption. However, the high humidity environment is quite common in the real nuclear industry and during the nuclear fuel reprocessing,^5^ which proposed the higher requirement for both the chemical stability and competitive iodine adsorption property of MOFs.^14^^,^^15^ Therefore, chemically stable high-performance MOFs for iodine capture and separation under humidity environment has attracted much research interest in the past few years.^16^^,^^17^^,^^18^^,^^19^ Notable studies include the work by Nenoff et al.,^16^ who combined the simulations and experiments to analyze the competitive adsorption behavior of Cu-BTC in a mixed-gas stream (∼1:1 ratio of I_2_:H_2_O vapor) at ambient pressure and 75°C. It was revealed that the Cu-BTC preferentially adsorbed I_2_ over water (selectivity of I_2_/H_2_O = 1.5 and iodine uptake ∼175 wt %). Thallapally and co-workers also reported the I_2_ adsorption research of SBMOF-1 and SBMOF-2 in the presence of humidity (33% RH and 43% RH) at room temperature.^17^ SBMOF-1 and SBMOF-2 showed 15 wt % and 35 wt % I_2_ uptakes, respectively. Zhang’s group conducted grand canonical Monte Carlo (GCMC) and density functional theory (DFT) methods to reveal the influence of H_2_O on different zeolitic imidazolate frameworks (ZIFs) during I_2_ adsorption.^18^ They found that the water had a negative impact on I_2_ uptake in hydrophilic materials due to the similar adsorption sites. Furthermore, besides the molecular form of iodine (I_2_), radioactive organic iodides mainly the methyl iodine (CH_3_I) are also the important components of the volatile iodine nuclides and have been considered to be captured.^20^^,^^21^ For instance, Li’s group constructed the MIL-101-Cr-TED and MIL-101-Cr-HMTA through post-synthetic modification to achieve the 35.2 wt % and 37.2 wt % uptake for CH_3_I at 150°C under humid conditions (RH = 81%).^22^ Yue et al. reported the generation of mesopores from ECUT-300, and enabled the corresponding adsorption capacity up to 0.85 g/g at 423 K under humid CH_3_I (RH = 50%).^23^ However, despite these advancements, the researches on MOFs for iodine adsorption in high humidity circumstance are still rare, much less the systematic comparison of the key factors (including the structural factors, hydrophilic properties, and modified functional groups) that influence the I_2_ and CH_3_I adsorption performance.

In this work, we first identified 21 MOFs with outstanding chemical and water stabilities based on previous researches,^24^^,^^25^ and conducted GCMC and DFT calculations to screen these high-performance MOFs for efficiently adsorbing and separating I_2_ and CH_3_I in humid air environments; next, the structural factors and hydrophilic properties were explored to investigate the effect on iodine capture; finally, different types of functional groups were grafted onto the MIL-53-Al to achieve the enhancement of iodine adsorption performances and then the underlying impact mechanism was also further analyzed.

## Results and discussion

### Computational screening of chemically stable MOFs

With reference to previous experimental studies, 21 representative MOFs with outstanding chemical and water stability have been initially selected.^24^^,^^25^ Relevant testing conditions and observations were shown in Table S1. GCMC simulations were performed to explore the adsorption and separation of gaseous I_2_ and CH_3_I in MOFs at conditions of 423 K and 1 bar, using the RASPA package.^26^ 423 K was the relevant operational temperature in the nuclear industry.^5^^,^^27^^,^^28^ To simulate the high humid environment during the actual reprocessing of the spent nuclear fuel, the mixed gas system was composed of 300 ppm I_2_ (or CH_3_I), 68.5% N_2_, 18.4% O_2_, and 12.2% H_2_O (achieving a relative humidity of 100%).^5^ Simulation details including the MOF structures, interatomic potentials, simulation parameter settings, and adsorption selectivity formula were provided in supplemental information.

The adsorption performance for I_2_ and CH_3_I, as well as the geometric properties of the 21 chemically stable MOFs, were listed in Table 1. The considered structural factors encompassed the pore limiting diameter (PLD, 3.01–28.26 Å), largest cavity diameter (LCD, 4.8–33.23 Å), void fraction (0.131–0.852), accessible surface area (S_cal_; 186.03–3594.65 m^2^/g), and pore volume (V_p-cal_; 0.087–1.744 cm^3^/g). The relatively high surface area and pore volume indicated these materials as great potential adsorbents for I_2_ and CH_3_I adsorption. The theoretically calculated surface area and pore volume (S_cal_ and V_p-cal_) could be roughly comparable to the values extracted from previous experiments (S_exp_ and V_p-exp_). Nonetheless, disparities existed, likely stemming from the presence of impurities or defects in the actual materials. Notably, for certain selected MOFs (e.g., UiO-66, UiO-66-NH_2_, JUC-110, etc.), the PLD was smaller than the kinetic diameter of CH_3_I molecules (4.23 Å), rendering them unsuitable for evaluating CH_3_I adsorption performance. It was evident that the uptake amounts of CH_3_I in MOFs were overall much lower than those of I_2_. This discrepancy arose from the comparatively inert chemical reactivity of CH_3_I,^9^ making the separation and immobilization of CH_3_I more challenging compared to that of elemental iodine.

**Table 1:** Adsorption performance and geometric properties of chemically stable MOFs

MOF	I_2_ uptake (cm^3^/g)	I_2_ selectivity	CH_3_I uptake (cm^3^/g)	CH_3_I selectivity	PLD (Å)	LCD (Å)	Void fraction	S_cal_ (m^2^/g)	S_exp_ (m^2^/g)	V_p-cal_ (cm^3^/g)	V_p-exp_ (cm^3^/g)
**Ni**_**3**_**(BTP)**_**2**_^29^	5.96	2.01 × 10^2^	0.39	12.58	9.75	14.59	0.373	1471.92	1026	0.496	0.44
**DUT-51-Hf**^30^	0.07	6.56	0.01	0.96	8.94	26.25	0.849	2354.42	1859	1.133	0.97
**DUT-51-Zr**^30^	0.10	3.68	0.014	0.53	9.04	26.32	0.852	3051.32	2335	1.478	1.08
**DUT-67-Zr**^31^	0.13	3.57	0.029	0.80	6.71	19.63	0.747	2079.13	1560	0.827	0.60
**MOF-545**^32^	0.15	9.20	0.038	2.38	28.26	33.23	0.826	3309.28	2490	1.744	1.73
**UiO-66**^33^	14.78	6.41 × 10^4^	/	/	3.85	8.58	0.506	1166.72	1000	0.418	0.44
**UiO-66-NH**_**2**_^34^	18.6	7.29 × 10^4^	/	/	3.54	7.29	0.473	936.36	873	0.377	0.379
**JUC-110**^35^	26.02	9.33 × 10^4^	/	/	3.53	6.67	0.353	478.78	456.3	0.271	
**Zn(1,3-BDP)**^36^	66.07	3.15 × 10^5^	0.75	1.38 × 10^3^	4.91	7.27	0.452	1192.7	820	0.404	
**NOTT-300**^37^	47.83	8.62 × 10^4^	2.22	1.81 × 10^3^	6.59	6.73	0.636	1379.08	1370	0.597	0.38
**PCN-224-Ni**^38^	0.12	23.12	0.02	4.44	14.48	22.95	0.792	3594.65	2600	1.597	1.59
**Al-PMOF**^39^	29.94	1.57 × 10^4^	5.56	3.01 × 10^3^	5.21	7.71	0.656	2198.85	1118	0.8	0.69
**SNU-80**^40^	22.51	2.95 × 10^4^	1.49	1.51 × 10^3^	7.06	7.58	0.400	1360.22	1035	0.469	0.43
**CALF-25**^41^	0.0065	78.79	0.021	2.57 × 10^2^	4.25	4.8	0.131	186.03	385	0.087	
**FMOF-1**^42^	0.089	8.22 × 10^3^	0.0053	50.62	5.44	6.19	0.392	816.06	810.5	0.222	0.32
**MIL-53-Al**^43^	49.85	1.60 × 10^5^	1.19	1.46 × 10^3^	6.18	6.32	0.602	1521.34	1235	0.614	0.54
**NU-1000**^44^	0.97	8.38 × 10^2^	0.06	55.55	27.12	29.12	0.813	3127.2	2577	1.574	1.45
**CAU-10**^31^	25.62	5.52 × 10^3^	/	/	3.61	6.01	0.380	749.48	600	0.33	0.26
**MIL-125-NH**_**2**_**-Ti**^45^	6.18	2.72 × 10^2^	0.3	13.29	4.42	11.25	0.579	1882.77	1469	0.665	0.60
**MOF-801-SC**^31^	9.70	4.61 × 10^4^	/	/	3.3	7.51	0.449	635.73	690	0.281	0.27
**ZIF-8**^46^	2.42	6.78 × 10^3^	/	/	3.01	11.77	0.446	1250.18	1291	0.466	0.53

### Relationships between structures and adsorption performance

**Figure 1:**
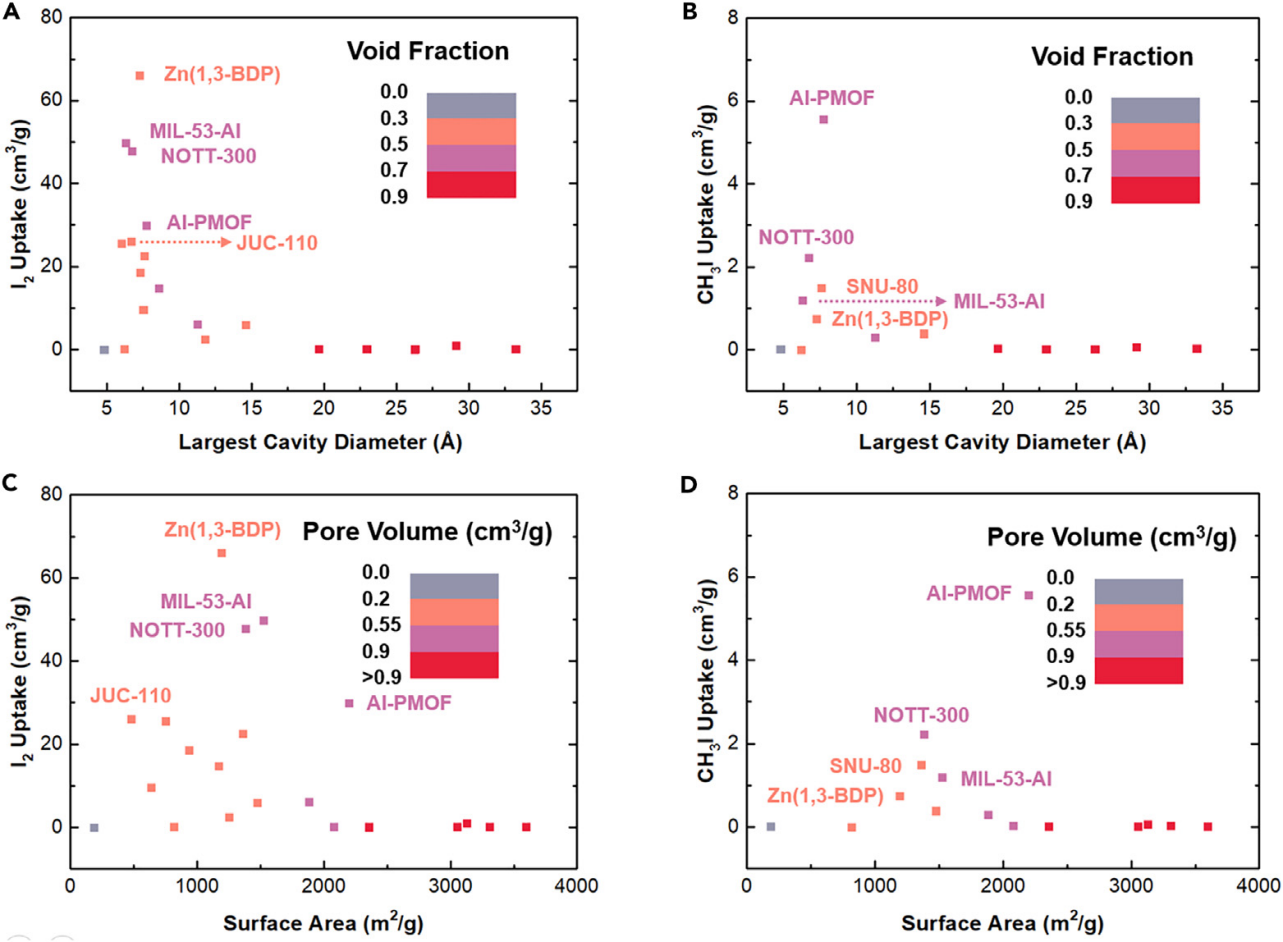
Relationships between structures and adsorption performance (A and B) (A) I_2_ and (B) CH_3_I uptake amounts vs. largest cavity diameter colored by void fraction. (C and D) (C) I_2_ and (D) CH_3_I uptake amounts vs. surface area colored by pore volume.

In order to further vividly reveal the effect of structural factors on adsorption performance, the structure-property relationships were illustrated in Figure 1. For I_2_ and CH_3_I adsorption, Figures 1A and 1B showed that the optimal LCD and void fraction were around 7.1 Å and at the range of 0.3–0.7, respectively; Figures 1C and 1D indicated that the optimal surface area and pore volume were at the range of 450–2230 m^2^/g and 0.2–0.9 cm^3^/g, respectively. The aforementioned four structural factors were correlative with each other (e.g., the larger LCD were more likely to induce the higher void fraction); but LCD exhibited more obvious and greater impact on adsorption performance, by causing the constricted porosity and compact interaction between the MOFs and iodine molecules. The aforementioned results were well consistent with the previous researches.^47^ We picked the several top-performing materials and marked their names in Figure 1. Zn(1,3-BDP), MIL-53-Al, NOTT-300, Al-PMOF, and JUC-110 presented the well-performing for I_2_ adsorption, exhibiting the large uptake amounts of 66.07, 49.85, 47.83, 29.94, and 26.02 cm^3^/g, respectively. The I_2_ selectivity of them also reached up to 3.15 × 10^5^, 1.60 × 10^5^, 8.62 × 10^4^, 1.57 × 10^4^, and 9.33 × 10^4^. The Al-PMOF, NOTT-300, SNU-80, MIL-53-Al, and Zn(1,3-BDP) showed the high CH_3_I uptake amounts and selectivity of 5.56 cm^3^/g (3.01×10^3^), 2.22 cm^3^/g (1.81 × 10^3^), 1.49 cm^3^/g (1.51 × 10^3^), 1.19 cm^3^/g (1.46 × 10^3^), and 0.75 cm^3^/g (1.38 × 10^3^), respectively.

**Figure 2:**
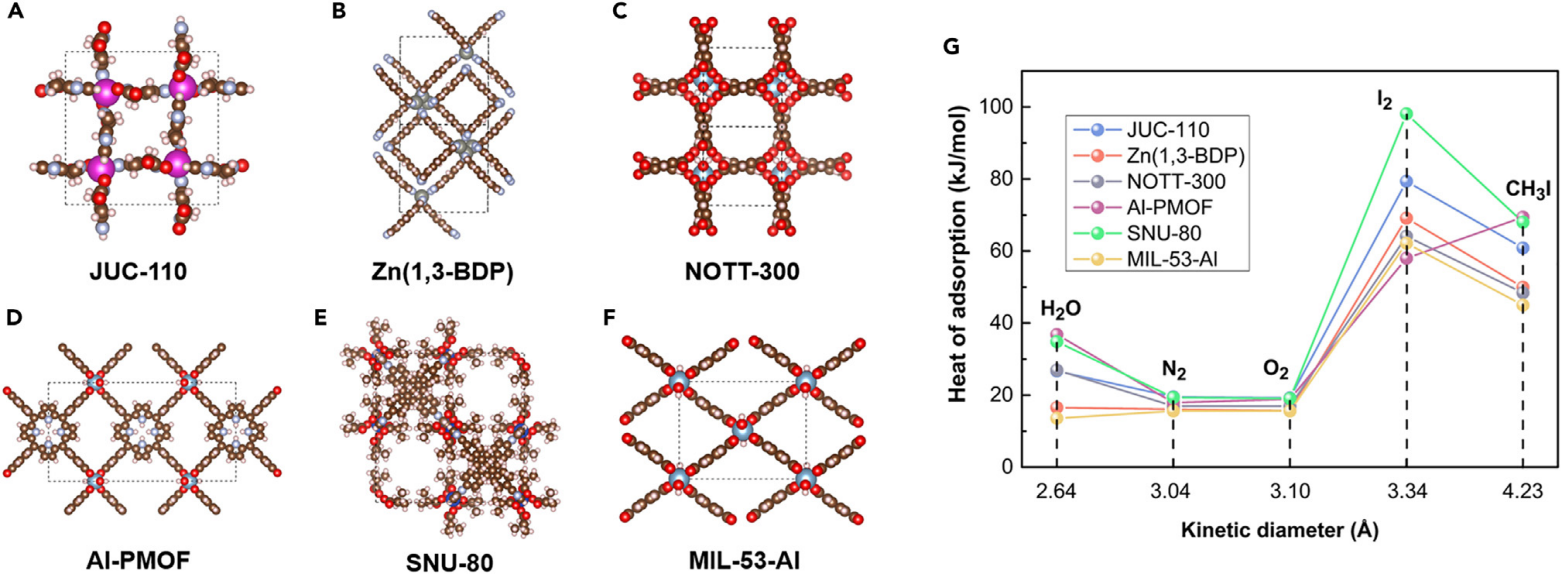
Structures and heat of adsorption of six top-performing materials (A‒F) Space-filling models of (A) JUC-110, (B) Zn(1,3-BDP), (C) NOTT-300, (D) Al-PMOF, (E) SNU-80, and (F) MIL-53-Al. (G) Isosteric heat of adsorption for guest gas molecule at infinite dilution in MOFs.

Figures 2A–2F plotted the porous structures of aforementioned six top-performing materials (i.e., JUC-110, Zn(1,3-BDP), NOTT-300, Al-PMOF, SNU-80, and MIL-53-Al). The isosteric heat of adsorption (Q_st_) of gaseous iodine (I_2_ and CH_3_I) and ambient atmosphere (H_2_O, N_2_ and O_2_) was shown in Figure 2G. The Q_st_ of I_2_ in SNU-80 outperformed all other selected MOFs, but its I_2_ uptake amount (22.51 cm^3^/g) was lower than other MOFs. The reason might lie in its high Q_st_ of H_2_O (34.8 kJ/mol), which led to the more drastic competitive adsorption between the H_2_O and I_2_ and therefore reduced I_2_ selectivity (2.95 × 10^4^) in SNU-80. Conversely, Zn(1,3-BDP) and MIL-53-Al with the lowest Q_st_ of H_2_O of 16.5 kJ/mol and 13.6 kJ/mol, exhibited the top-two I_2_ uptake amounts and selectivity. Consequently, it could be concluded that the Q_st_ of H_2_O had a significant impact on I_2_ adsorption due to competitive adsorption in MOFs. However, compared to I_2_ adsorption, the influence of Q_st_ of H_2_O on CH_3_I adsorption was not so remarkable; whereas the Q_st_ of CH_3_I played the main role. Al-PMOF with the highest Q_st_ of CH_3_I of 69.4 kJ/mol, owned relatively high Q_st_ of H_2_O (36.8 kJ/mol), but its CH_3_I uptake amounts (5.56 cm^3^/g) and selectivity (3.01 × 10^3^) were still the highest among selected MOFs. Similar characteristics could also be found in SNU-80 for CH_3_I adsorption. Furthermore, the adsorption isotherms of the pure gaseous I_2_, CH_3_I, H_2_O, N_2_, and O_2_ in JUC-110, Zn(1,3-BDP), NOTT-300, Al-PMOF, SNU-80, and MIL-53-Al were also simulated (Figure S1). All of the materials exhibited much better adsorption performance for I_2_ and CH_3_I than humid air atmosphere (N_2_, O_2_, and H_2_O). MIL-53-Al and Zn(1,3-BDP) exhibited the best hydrophobicity in adsorption isotherms, consistent with the ranking of Q_st_ of H_2_O.

### Functional tuning of MIL-53-Al

**Figure 3:**
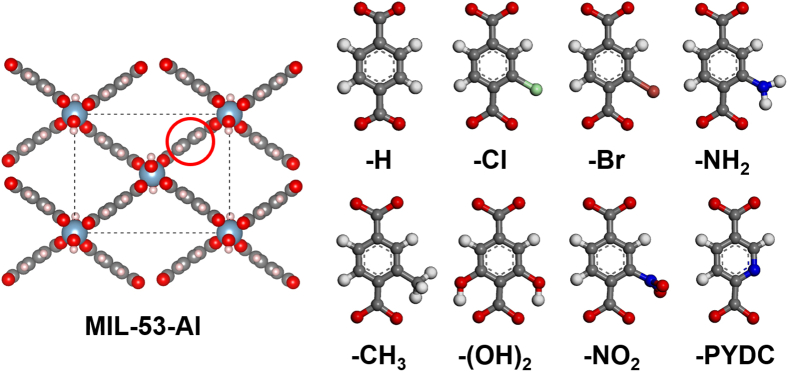
The structures of MIL-53-Al grafted with different functional groups (color scheme: Al, cyan; C, gray; O, red; H, white; N, blue; Cl, green; Br, brown).

In pursuit of the better adsorption performances, MIL-53-Al was picked out to tune the surface functionality, due to its excellent comprehensive performance including the high I_2_ and CH_3_I uptake amounts, extraordinary hydrophobicity (with the lowest Q_st_ of H_2_O) and the most critically—facile modification. As shown in Figure 3, different types of functional groups were grafted onto the benzene ring of MIL-53-Al to get MIL-53-Al-X series, in which MIL-53-Al-H represents the pristine MIL-53-Al without functionalization. After the fully structural relaxation using DFT calculations, theoretical geometric properties of MIL-53-Al-X series were listed in Table 2. The adsorption isotherms of the pure gaseous I_2_, CH_3_I, N_2_, O_2_, and H_2_O in MIL-53-Al-X series at 423 K and 0–1 bar were simulated and plotted in Figure S2. The I_2_ and CH_3_I adsorption isotherms in the MIL-53-Al-X series featured type I micropore filling adsorption mechanism with the uptake saturated at relatively low pressure. The low H_2_O uptake amount (below 3 cm^3^/g even at 1 bar of pure H_2_O) shown in H_2_O adsorption isotherms indicated the MIL-53-Al-X series as the good hydrophobic materials.

**Table 2:** Adsorption performance and geometric properties of functionalized MIL-53-Al-X

MIL-53-Al-X	I_2_ uptake (cm^3^/g)	I_2_ selectivity	CH_3_I uptake (cm^3^/g)	CH_3_I selectivity	PLD (Å)	LCD (Å)	Void fraction	S_cal_ (m^2^/g)	V_p-cal_ (cm^3^/g)
**-H**	49.85	1.60×10^5^	1.19	1.46×10^3^	6.18	6.32	0.602	1521.34	0.614
**-Cl**	44.96	2.80×10^5^	1.51	2.23×10^3^	5.22	6.23	0.529	1154.76	0.463
**-Br**	36.72	3.75×10^5^	1.22	2.38×10^3^	5.44	5.81	0.499	920.38	0.369
**-NH**_**2**_	50.65	2.92×10^5^	1.81	2.36×10^3^	5.31	6.32	0.535	1273.53	0.509
**-CH**_**3**_	49.74	4.99×10^5^	2.8	3.62×10^3^	4.89	6.08	0.492	1192.24	0.47
**-(OH)**_**2**_	44.51	2.62×10^5^	1.45	2.09×10^3^	5.04	5.68	0.529	1194.1	0.468
**-NO**_**2**_	42.66	2.72×10^5^	1.45	2.19×10^3^	5.41	5.76	0.542	1156.43	0.455
**-PYDC**	41.07	1.08×10^5^	0.73	9.89×10^2^	6.19	6.48	0.607	1543.52	0.617

**Figure 4:**
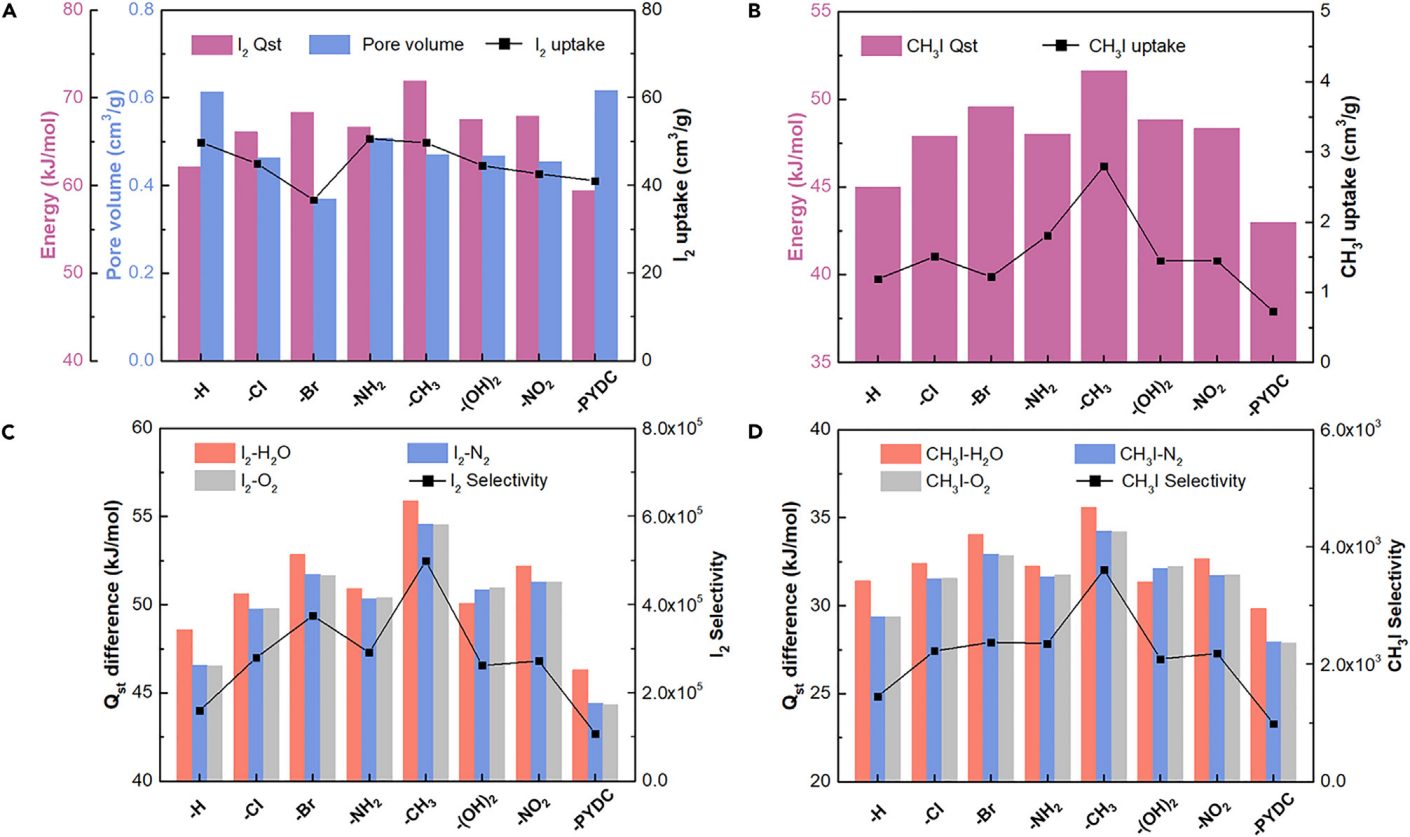
Adsorption performance studies of functionalized MIL-53-Al-X series (A and B) Correlations between the Q_st_ (and pore volume) and adsorption capacity of (A) I_2_ and (B) CH_3_I in MIL-53-Al-X series. (C and D) Correlations between Q_st_ difference of (C) I_2_ and (D) CH_3_I against other adsorbates and adsorption selectivity in MIL-53-Al-X series.

Subsequently, adsorption simulations in the mixed gas system were applied to elucidate the trace I_2_ and CH_3_I adsorption performance. The mixed gas composition was set as the same conditions as before in the investigations of initial 21 chemically robust MOFs. The adsorption performance and geometric properties of functionalized MIL-53-Al-X were shown in Table 2; and the isosteric heat of adsorption for guest gas molecule was plotted in Figure S3. It was noteworthy that the Q_st_ of H_2_O (ranging from 13.1 kJ/mol to 17.5 kJ/mol) in functionalized MIL-53-Al-X series maintained the low level with relatively small energy differences, and therefore had no longer played the obvious role in I_2_ uptake amounts. This phenomenon was different from the previous six top-performing materials (i.e., JUC-110, Zn(1,3-BDP), NOTT-300, Al-PMOF, SNU-80, and MIL-53-Al). Table 2 exhibited that the functionalization of the polarized functional groups did enhance the I_2_ selectivity (except the MIL-53-Al-PYDC), but the I_2_ adsorption amounts did not have the obvious enhancement. For researching the reason, we analyzed the influence of different structure factors of MIL-53-Al-X series on I_2_ adsorption capacity in Figure S4, in which an obvious correlation was observed between the pore volume and I_2_ uptake amount. So, it was attributed to the smaller pore volume and bigger steric hindrance that limited the I_2_ uptake in functionalized MIL-53-Al-X series, despite the enhanced the I_2_ selectivity. Figure 4A illustrated that the pore volumes (0.37–0.62 cm^3^/g) were a major determining factor, whereas the Q_st_ of I_2_ (59.4–71.9 kJ/mol) played a relatively minor role in influencing I_2_ uptake. For instance, MIL-53-Al-Cl and MIL-53-Al-Br had the relatively high I_2_ selectivity of 2.80×10^5^ and 3.75×10^5^, considerably higher than that of unfunctionalized MIL-53-Al (1.60×10^5^, 49.85 cm^3^/g). However, their small pore volumes (0.463 cm^3^/g and 0.369 cm^3^/g) resulted in the lower I_2_ adsorption amounts of 44.96 cm^3^/g and 36.72 cm^3^/g, respectively. Conversely, MIL-53-Al-NH_2_ and MIL-53-Al-CH_3_ with relatively large pore volumes (0.509 cm^3^/g and 0.470 cm^3^/g) exhibited the top-two I_2_ uptake amounts of 50.65 cm^3^/g and 49.74 cm^3^/g, respectively. Regarding MIL-53-Al-PYDC, the largest pore volume (0.617 cm^3^/g) was balanced with the lowest I_2_ affinity (59.4 kJ/mol), resulting in a mediocre CH_3_I uptake amount of 41.07 cm^3^/g. Additionally, the good correlation shown in Figure 4B demonstrated that the Q_st_ difference of I_2_ against other adsorbates governed the I_2_ adsorption selectivity in MIL-53-Al-X series.

With regard to CH_3_I adsorption, as shown in Table 2 and Figures 4C and 4D, the modified functional groups significantly enhanced the CH_3_I adsorption performances, including both CH_3_I uptake amounts and selectivity. The CH_3_I uptake amounts and selectivity were positively correlated with the Q_st_ of CH_3_I, indicating the affinity for CH_3_I played a major role in CH_3_I uptake in MIL-53-Al-X series. The impact of the functionalization-induced reduction in pore volume was nearly negligible (Figure S5). MIL-53-Al-CH_3_, exhibiting the highest Q_st_ for CH_3_I of 71.95 kJ/mol, achieved the highest CH_3_I uptake amount of 2.8 cm^3^/g, which was more than twice that of the pristine MIL-53-Al (1.19 cm^3^/g); followed by the MIL-53-Al-NH_2_ with the CH_3_I uptake amount of 1.81 cm^3^/g.

**Figure 5:**
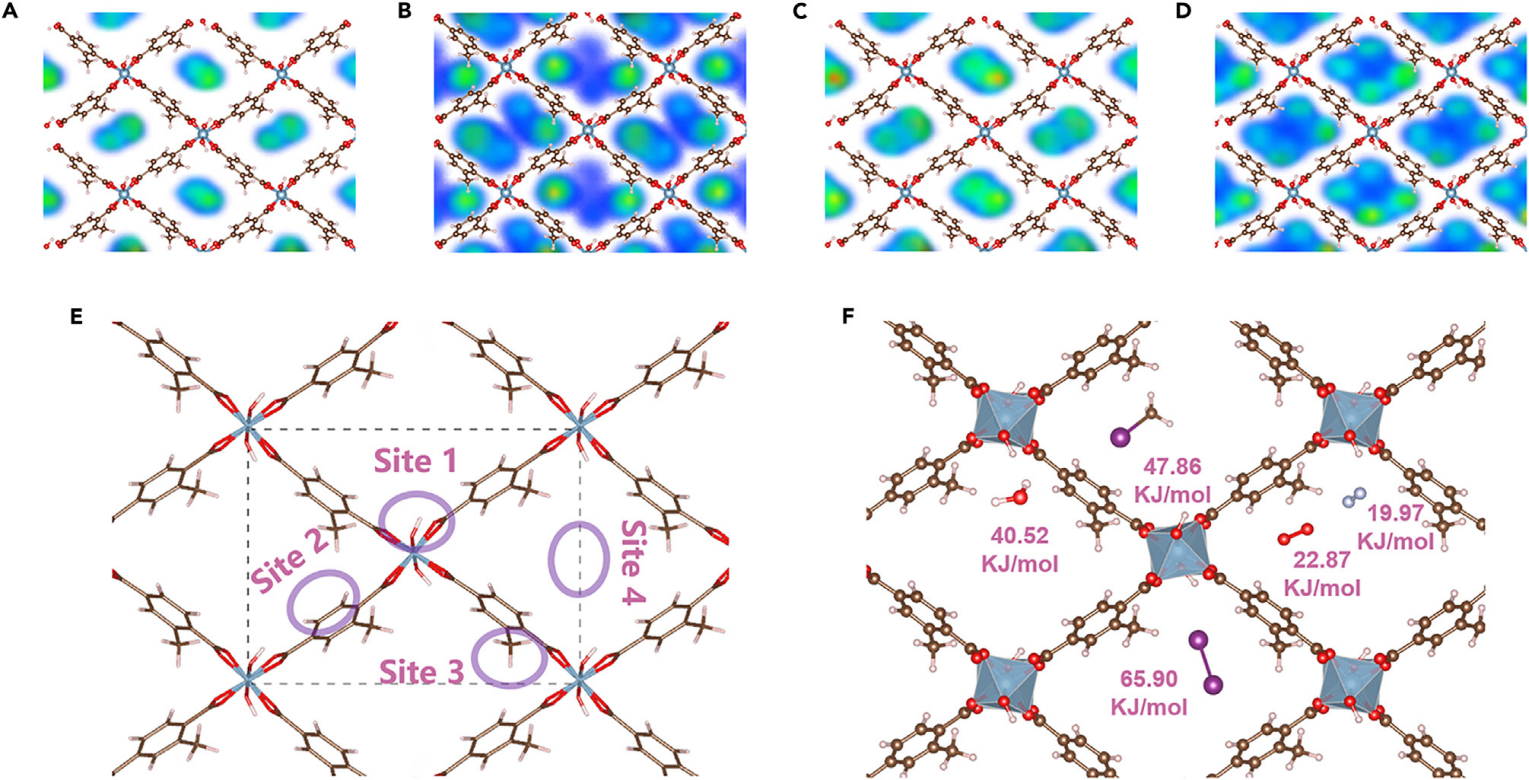
Adsorption location studies in MIL-53-Al-CH_3_ (A and B) Adsorption density plot pictures of (A) I_2_ and (B) H_2_O during competitive I_2_ adsorption. (C and D) Adsorption density plot pictures of (C) CH_3_I and (D) H_2_O during competitive CH_3_I adsorption. (E and F) (E) Simulated adsorption locations for DFT calculations and (F) DFT-optimized optimal geometric positions of different molecules in the MIL-53-Al-CH_3_ pore channel.

Figures 5A–5D presented the GCMC-simulated adsorption density plots of I_2_, CH_3_I, and H_2_O during the competitive adsorption. It could be observed that the I_2_ and CH_3_I molecules were primarily concentrated near the center of pore channels, whereas the H_2_O molecules were mainly located near the backbones of the framework. The above differences of the density contour shapes could be attributed to the varying uptake amounts and molecule sizes. The larger space near the center of pore channels allowed for accommodating a greater number of larger molecules (e.g., I_2_ and CH_3_I). For H_2_O molecules, the sites near the Al clusters or methyl appeared to exhibit a higher affinity, likely due to the strong hydrogen bonding interactions. To understand the aforementioned phenomenon at the molecular scale, DFT calculations were applied to determine the corresponding binding energies between the MOF and individual molecule (I_2_, CH_3_I, N_2_, O_2_, or H_2_O) at four possible adsorption sites, depicted in Figure 5E: near the Al cluster, above the benzene ring, around the methyl (-CH_3_), and at the center of the pore channel. The binding energies were detailed in Table S4, and the optimal adsorption situations with the highest binding energies for each molecule were shown in Figure 5F. The optimal adsorption positions for I_2_ (65.90 kJ/mol) and CH_3_I (47.86 kJ/mol) were slightly off-center from the channel; the smaller N_2_ and O_2_ molecules could be adsorbed near the Al clusters, benzene ring and methyl due to their smaller steric hindrance; the adsorption situations for H_2_O were complex: Al clusters were the optimal adsorption sites with the highest binding energy of 40.52 kJ/mol, followed by the methyl (34.16 kJ/mol), the benzene ring (23.25 kJ/mol), and the center of the pore channel (12.45 kJ/mol). Importantly, the hydroxyl functional groups connected to Al clusters played a significant role in hydrogen bonding interactions to achieve a high binding energy. Additionally, the binding energies of pristine MIL-53-Al at different adsorption sites were also calculated (Table S5), conforming the methyl functionalization really enhanced the adsorption of I_2_ and CH_3_I. These DFT calculations also confirmed the validity of the GCMC simulation results.

### Conclusions

In summary, we have performed GCMC and DFT calculations to screen 21 experimentally confirmed chemically stable MOFs and identified MIL-53-Al as a promising iodine adsorbent under high humidity circumstances. Structural factors have a significant effect on adsorption performance for both I_2_ and CH_3_I; whereas hydrophilicity had a more pronounced impact on I_2_ adsorption behaviors compared to CH_3_I. The introduction of polar functional groups proved to substantially enhance the iodine adsorption selectivity by promoting the adsorption affinity. However, this enhancement came at the cost of reduced pore volume, particularly affecting the uptake capacity for I_2_ adsorption. Overall, MIL-53-Al-CH_3_ outperformed other MOFs, exhibiting notably improved CH_3_I uptake (2.8 cm^3^/g) and selectivity (3.62×10^3^), while simultaneously maintaining excellent I_2_ uptake (49.74 cm^3^/g) and selectivity (4.99×10^5^). Moreover, at the molecular level, it was revealed that I_2_ and CH_3_I molecules were primarily adsorbed near the channel center in MIL-53-Al-CH_3_; whereas other competitive gas molecules tended to be located around the backbones of the framework. Generally, this work has provided a feasible screening and design approach for promising high-performance iodine adsorbents in nuclear waste managements under high humidity conditions.

### Limitations of the study

Our work has theoretically screened and studied the chemically stable high-performance MOF as iodine adsorbents in humid environments. However, the experimental investigation and validation on the iodine adsorption performance needs to be further explored.

## STAR★Methods

### Key resources table

**Table:** 

REAGENT or RESOURCE	SOURCE	IDENTIFIER
**Software and algorithms**		

RASPA	Personally, by David Dubbeldam et al.	https://iraspa.org/raspa/
VASP 5.4.4	VASP Software GmbH	https://www.vasp.at/
Zeo++	Personally, by Maciej Haranczyk et al.	http://www.zeoplusplus.org/
VESTA	Personally, by Koichi Momma	https://jp-minerals.org/vesta/en/
Origin	OriginLab	https://www.originlab.com/

### Resource availability

#### Lead contact

Further information and requests for resources should be directed to and will be fulfilled by the lead contact, Guangcun Shan (gshan2-c@my.cityu.edu.hk).

#### Materials availability

This study did not generate new unique reagents.

#### Data and code availability

• The published article includes all datasets generated or analyzed during this study.
• This paper does not report original code.
• Any additional information required to reanalyze the data reported in this paper is available from the lead contact upon request.

### Method details

#### MOF structures

The guest-free crystalline structures of MOFs were obtained from Cambridge Structural Database (CSD). Considering the well-known breathing effect of MIL-53-Al, whose pore size was tunable and could undergo great changes, the high temperature form of MIL-53-Al (MIL-53-Al-ht) was used to conform to the high temperature environment of 423 K.^28^^,^^48^ The structure features of the aforementioned MOFs, including pore limiting diameter (PLD) and largest cavity diameter (LCD), were calculated using the open-source package Zeo++.^49^

#### Interatomic potentials

In this work, the nonbonded interactions were described with Lennard-Jones (LJ) and Coulombic potentials, and the cross-interaction potential parameters were calculated by Lorentz-Berthelot mixing rules. The guest I_2_ molecules had a kinetic diameter of 3.34 Å and were modeled as spherical model, the LJ potential parameters of which were taken from the literature and derived from the viscosity calculation of pure I_2_.^50^ The CH_3_I molecules with a kinetic diameter of 4.23 Å were represented by an explicit all-atom model, having LJ interaction site and partial atomic charge placed on each atom, and the potential parameters were taken from the work of Crone-Munzebrock and Doge.^51^ The H_2_O molecules (kinetic diameter of 2.64 Å) were mimicked by the transferable intermolecular potential (TIP3P) model (*r*_OH_ = 0.9527 Å and *θ*_∠HOH_ = 104.52°),^52^ which has been proved to accurately describe the hydrogen bonding characteristics and successfully used in previous water-adsorption simulation works.^53^ Both N_2_ and O_2_ molecules (kinetic diameter of 3.04 Å and 3.10 Å, respectively) were modeled by a three-site model, in which the mass was centered in molecules, and the N or O atoms carried the partial atomic charges and the LJ interaction site.^54^^,^^55^ In terms of MOFs, an atomistic representation was employed with universal force field (UFF) applied to assign LJ potential parameters.^56^ The potential parameters of the aforementioned guest molecules and MOFs were respectively listed in Tables S2 and S3. In addition, to accurately address the electrostatic interaction, atomic charges of MOFs were computed using the high-quality density-derived electrostatic charges (DDECs) method.^57^

#### Grand canonical Monte Carlo simulations

The whole GCMC simulations consisted of an initial equilibrium run of 1×10^6^ Monte Carlo (MC) cycles and a subsequent production run of 2 × 10^6^ MC cycles. In every cycle, there were six types of trials for molecules with the same probability: translation, rotation, reinsertion, identity change, creation and deletion. All the MOFs structures were treated as rigid frameworks with periodic boundaries; when necessary, supercells were applied to ensure the system size to be twice longer than the cutoff distance (12 Å). In addition, the selectivity of I_2_ and CH_3_I during adsorption was calculated as the following equation:^8^^,^^28^

$${selectivity}_{\text{iodine}}=\frac{X_{\text{iodine}}/Y_{\text{iodine}}}{X_{\text{others}}/Y_{\text{others}}}$$

where $X_{\text{iodine}}$ and $Y_{\text{iodine}}$ denoted the uptake amounts and gas phase concentration of targeted gas iodine (I_2_ or CH_3_I), respectively; $X_{\text{others}}$ and $Y_{\text{others}}$ were the uptake amounts and gas phase concentration of other gas components (N_2_, O_2_ and H_2_O).

#### Density functional theory calculations

DFT calculations were carried out with the Perdew-Burke-Ernzerhof (PBE) functional in the generalized gradient approximation (GGA) using the Vienna ab initio simulation package (VASP).^58^^,^^59^^,^^60^^,^^61^ The cut-off energy for the plane wave basis was set as 500 eV. DFT-D3(BJ) dispersion corrections were included to consider the van der Waals interactions.^62^^,^^63^ Besides, the binding energy between the MOF and guest gas molecule was determined through the following equation:

$$\Delta E=E_{\text{coordination}}-E_{\text{MOF}}-E_{\text{guest}}$$

where $E_{\text{coordination}}$ stood for the optimized energy of the coordinated structures after fully structure relaxation, $E_{\text{MOF}}$ and $E_{\text{guest}}$ were the energy of the individual MOF and guest molecule, respectively.^19^

## Supplemental information

Document S1. Figures S1‒S5 and Tables S1‒S5
