# Supplementary material for: Computational screening and functional tuning of chemically stable metal organic frameworks for I2/CH3I capture in humid environments
Source: iScience. 2024 Feb 6;27(3):109096. doi: 10.1016/j.isci.2024.109096 (PMC10877947; doi:10.1016/j.isci.2024.109096)
Supplement: Document S1. Figures S1‒S5 and Tables S1‒S5 [file mmc1.pdf]

iScience, Volume *27*

Supplemental information

Computational screening and functional tuning of chemically stable metal organic frameworks for I_2/CH_3I capture in humid environments

Haoyi Tan and Guangcun Shan

Table S1. Chemical stability testing of selected MOFs in previous experimental studies. Related to Table 1.

MOF	Testing condition	Observation
**$Ni_3(BTP)_2$**[1]	Boiling water, 2 weeks	No change in PXRD and BET surface area
**DUT-51-Hf**[2]	Soaked in water, 12 h	Slight reduction of BET surface area
**DUT-51-Zr**[2]	Soaked in water, 12 h	Slight reduction of BET surface area
**DUT-67-Zr**[3]	Soaked in water, 24 h	No change in PXRD
**MOF-545**[4]	Soaked in water, 12 h	After reactivation, "the crystallinity and porosity of the materials were completely recovered."
**UiO-66**[5]	Boiling water, 15 h	No change in PXRD
**UiO-66-NH_2**[6]	Soaked in water, 2 h	No change in PXRD
**JUC-110**[7]	Boiling water, 10 days	"JUC-110 retains the crystalline structure"
**Zn(1,3-BDP)**[8]	Boiling water, 3 days	"The structure is fully retained"
**NOTT-300**[9]	Water vapour, 90-100°C, 1 h/4 cycles	No apparent loss of uptake capacity
**PCN-224-Ni**[10]	Water/acid/base, 24 h	No change in PXRD and BET surface area
**Al-PMOF**[11]	Water/acid/base, 7 days	No change in PXRD
**SNU-80**[12]	Soaked in water, 7 days	No change in PXRD
**CALF-25**[13]	Water vapour, 353 K, 24 h	No change in PXRD or decrease of CO_2 uptake
**FMOF-1**[14]	Soaked in water, several days	No change in single crystal XRD
**MIL-53-Al**[15]	Water/acid/base, 7 days	No change in PXRD
**NU-1000**[16]	Soaked in water, 1 day	No change in PXRD
**CAU-10**[17]	Water/acid/base, 18 h	No change in PXRD
**MIL-125-NH_2-Ti**[18]	Water, 2 days	No change in PXRD
**MOF-801-SC**[19]	2 water adsorption cycles	Little change in water uptake properties
**ZIF-8**[20]	Water vapour and acid, 3 days	No change in PXRD

Table S2. Potential parameters of guest molecules. Related to Table 1.

Molecules	site	σ (Å)	ε/k_B (K)	Charges (*e*)
**I_2**	I	4.982	550	0
	C	3.40	51.22	-0.02
**CH_3I**	H	2.20	10.01	0.052
	I	4.12	324.06	-0.136
**H_2O**	O	3.151	76.42	-0.834
	H	0	0	0.417
**N_2**	N	3.31	36.0	-0.482
	N_2_center	0	0	0.964
**O_2**	O	3.02	49.0	-0.112
	O_2_center	0	0	0.224

Table S3. Potential parameters of MOFs. Related to Table 1.

Atoms	σ (Å)	ε/k_B (K)	Atoms	σ (Å)	ε/k_B (K)
**Ag**	2.80	18.11	**Zr**	2.78	34.72
**Al**	4.01	254.09	**C**	3.43	52.83
**Ba**	3.30	183.15	**H**	2.57	22.14
**Cd**	2.54	114.72	**O**	3.12	30.19
**Cu**	3.11	2.52	**N**	3.26	34.72
**Fe**	2.59	6.54	**P**	3.69	153.46
**Hf**	2.80	36.23	**S**	3.59	137.86
**Ni**	2.52	7.55	**F**	3.00	25.16
**Ti**	2.83	8.55	**Cl**	3.52	114.21
**Zn**	2.46	62.39	**Br**	3.73	126.29

Table S4. The binding energies (KJ/mol) of MIL-53-Al-CH_3 at different adsorption sites (The optimal adsorption situations are marked with the underline). Related to Figure 5.

Molecules	Site 1	Site 2	Site 3	Site 4
**I_2**	**<u>65.90</u>**	61.36	59.15	59.63
**CH_3I**	46.89	**<u>47.86</u>**	43.03	39.56
**N_2**	**<u>19.97</u>**	16.11	19.68	11.00
**O_2**	22.29	**<u>22.87</u>**	21.90	14.09
**H_2O**	**<u>40.52</u>**	23.25	34.16	12.45

Table S5. The binding energies (KJ/mol) of pristine MIL-53-Al at different adsorption sites (The optimal adsorption situations are marked with the underline). Related to Figure 5.

Molecules	Site 1	Site 2	Site 3	Site 4
**I_2**	**<u>58.86</u>**	52.97	/	47.47
**CH_3I**	**<u>45.35</u>**	39.66	/	35.51
**N_2**	**<u>17.46</u>**	14.47	/	9.46
**O_2**	**<u>19.49</u>**	19.01	/	12.83
**H_2O**	**<u>38.79</u>**	16.69	/	7.14

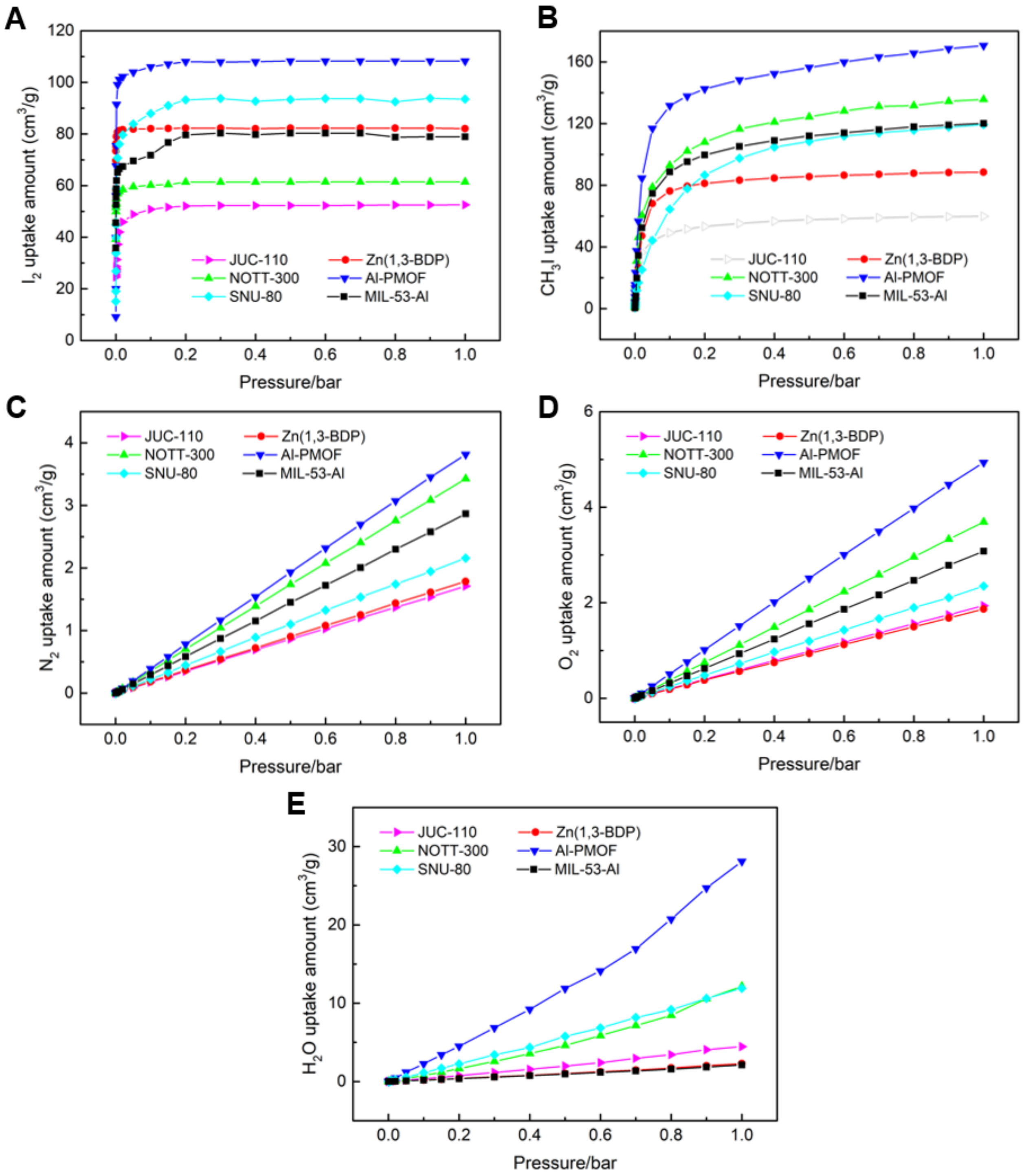

Figure S1. Adsorption isotherms in the JUC-110, Zn(1,3-BDP), NOTT-300, Al-PMOF, SNU-80 and MIL-53-Al at 423K and 0~1 bar pressure of pure (A) I_2; (B) CH_3I; (C) N_2; (D) O_2; (E) H_2O. Related to Figure 2.

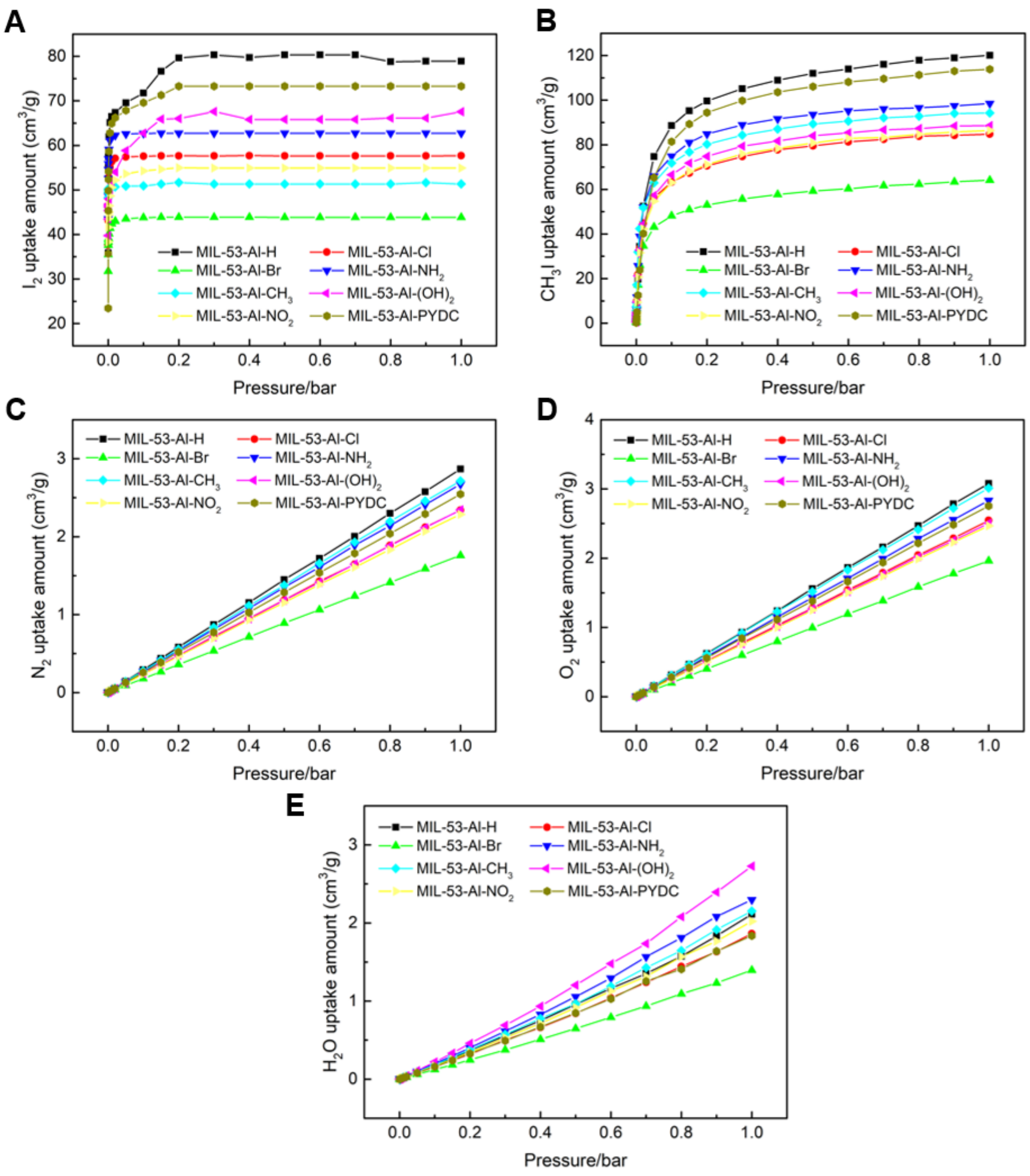

Figure S2. Adsorption isotherms in the MIL-53-Al-X at 423K and 0~1 bar pressure of pure (A) I_2; (B) CH_3I; (C) N_2; (D) O_2; (E) H_2O. Related to Figure 3.

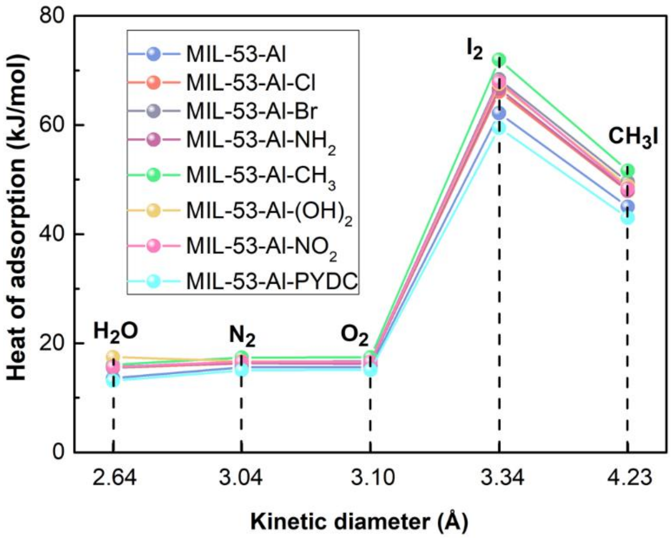

Figure S3. Isosteric heat of adsorption for guest gas molecule at infinite dilution in MIL-53-Al-X series. Related to Figure 4.

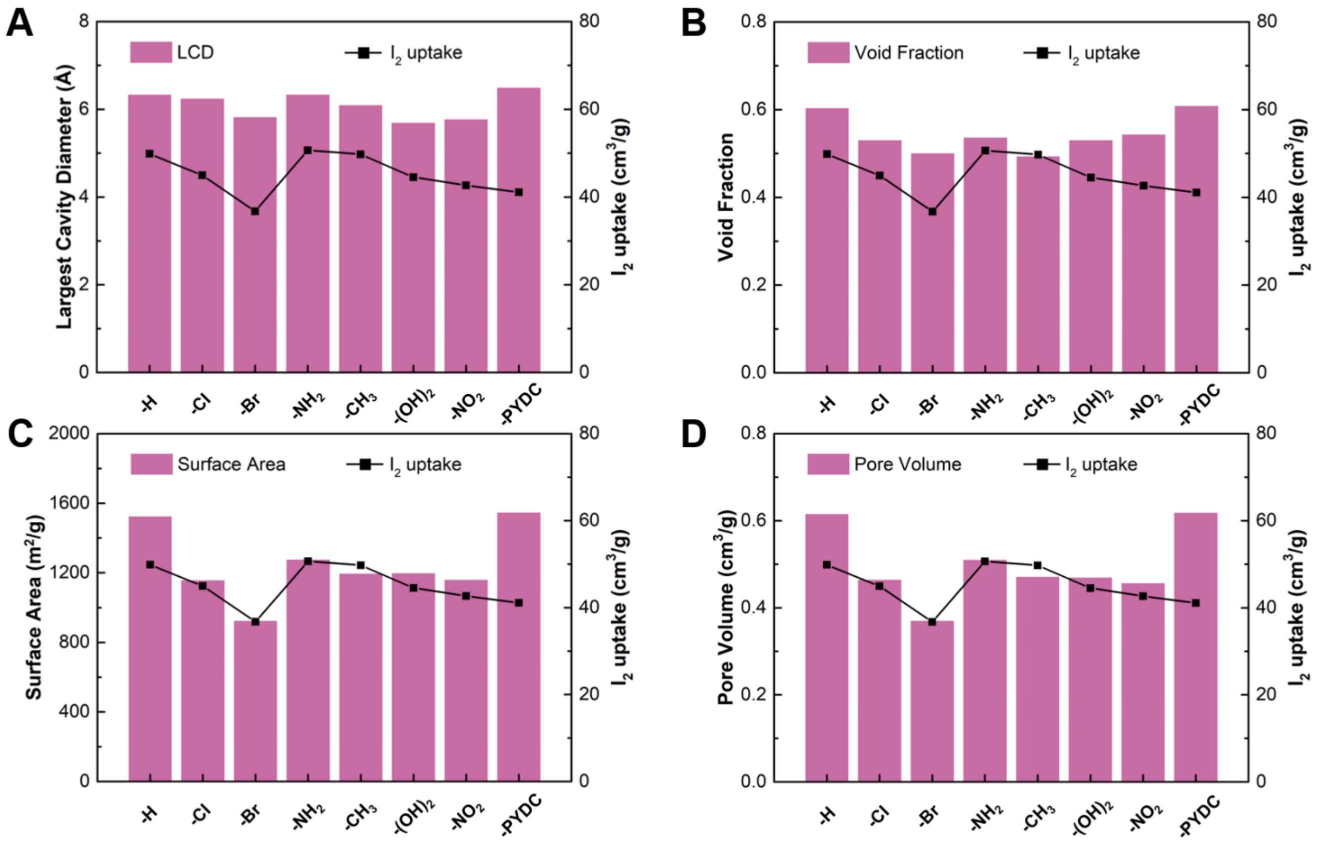

Figure S4. Correlations between the structure factors and adsorption capacity of I_2 in MIL-53-Al-X series. Related to Figure 4.

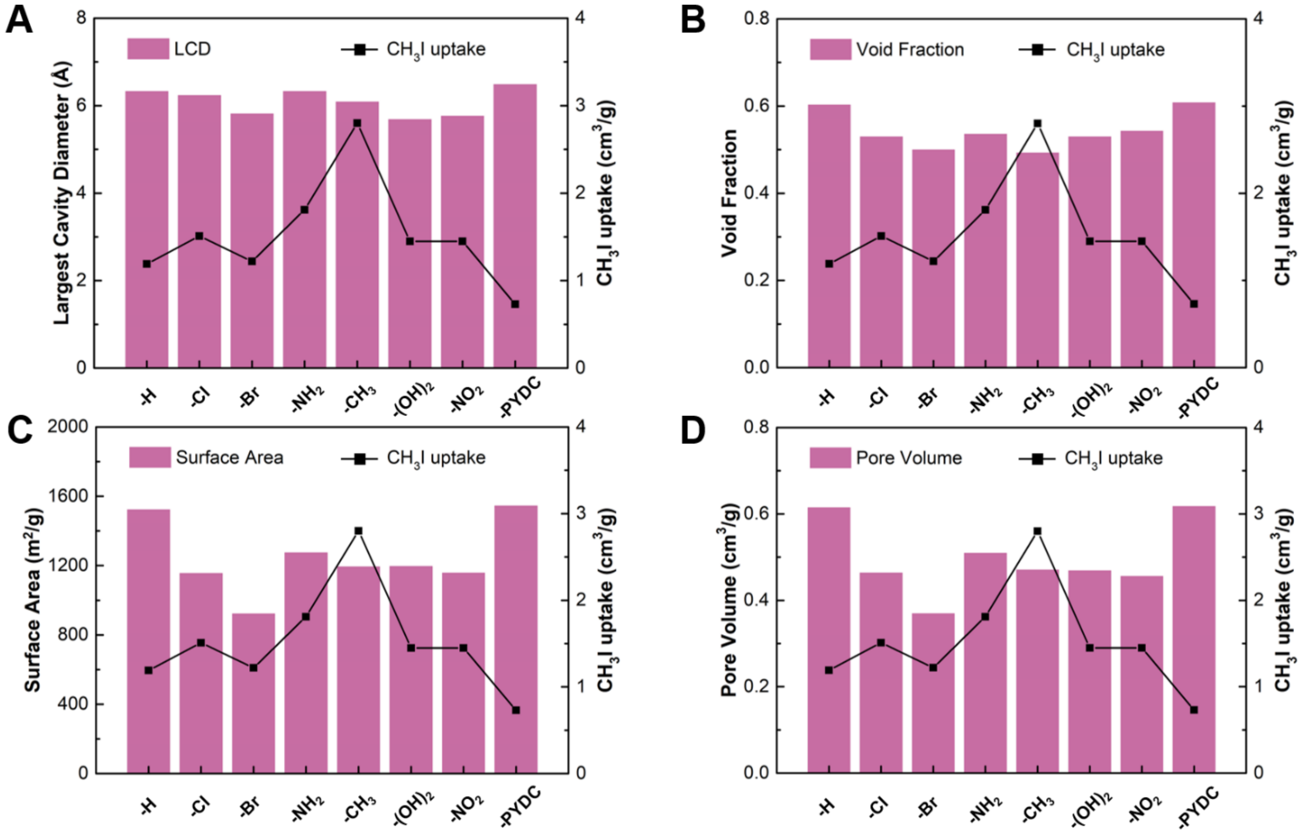

Figure S5. Correlations between the structure factors and the adsorption capacity of CH_3I in MIL-53-Al-X series. Related to Figure 4.

Supplemental references

[S1] Colombo, V., Galli, S., Choi, H.J., Han, G.D., Maspero, A., Palmisano, G., Masciocchi, N., and Long, J.R. (2011). High thermal and chemical stability in pyrazolate-bridged metal–organic frameworks with exposed metal sites. Chem. Sci. *2*, 1311-1319.

[S2] Bon, V., Senkovskyy, V., Senkovska, I., and Kaskel, S. (2012). Zr(IV) and Hf(IV) based metal-organic frameworks with reo-topology. Chem. Commun. *48*, 8407-8409.

[S3] Bon, V., Senkovska, I., Baburin, I.A., and Kaskel, S. (2013). Zr- and Hf-Based Metal–Organic Frameworks: Tracking Down the Polymorphism. Cryst. Growth Des. *13*, 1231-1237.

[S4] Morris, W., Volosskiy, B., Demir, S., Gandara, F., McGrier, P.L., Furukawa, H., Cascio, D., Stoddart, J.F., and Yaghi, O.M. (2012). Synthesis, structure, and metalation of two new highly porous zirconium metal-organic frameworks. Inorg. Chem. *51*, 6443-6445.

[S5] Guillerm, V., Ragon, F., Dan-Hardi, M., Devic, T., Vishnuvarthan, M., Campo, B., Vimont, A., Clet, G., Yang, Q., Maurin, G., et al. (2012). A series of isoreticular, highly stable, porous zirconium oxide based metal-organic frameworks. Angew. Chem. *51*, 9267-9271.

[S6] Kandiah, M., Nilsen, M.H., Usseglio, S., Jakobsen, S., Olsbye, U., Tilset, M., Larabi, C., Quadrelli, E.A., Bonino, F., and Lillerud, K.P. (2010). Synthesis and Stability of Tagged UiO-66 Zr-MOFs. Chem. Mater. *22*, 6632-6640.

[S7] Borjigin, T., Sun, F., Zhang, J., Cai, K., Ren, H., and Zhu, G. (2012). A microporous metal-organic framework with high stability for GC separation of alcohols from water. Chem. Commun. *48*, 7613-7615.

[S8] Choi, H.J., Dincă, M., Dailly, A., and Long, J.R. (2010). Hydrogenstorage in water-stable metal–organic frameworks incorporating 1,3- and 1,4-benzenedipyrazolate. Energy Environ. Sci. *3*, 117-123.

[S9] Yang, S., Sun, J., Ramirez-Cuesta, A.J., Callear, S.K., David, W.I., Anderson, D.P., Newby, R., Blake, A.J., Parker, J.E., Tang, C.C., and Schroder, M. (2012). Selectivity and direct visualization of carbon dioxide and sulfur dioxide in a decorated porous host. Nat. Chem. *4*, 887-894.

[S10] Feng, D., Chung, W.C., Wei, Z., Gu, Z.Y., Jiang, H.L., Chen, Y.P., Darensbourg, D.J., and Zhou, H.C. (2013). Construction of ultrastable porphyrin Zr metal-organic frameworks through linker elimination. J. Am. Chem. Soc. *135*, 17105-17110.

[S11] Fateeva, A., Chater, P.A., Ireland, C.P., Tahir, A.A., Khimyak, Y.Z., Wiper, P.V., Darwent, J.R., and Rosseinsky, M.J. (2012). A water-stable porphyrin-based metal-organic framework active for visible-light photocatalysis. Angew. Chem. *51*, 7440-7444.

[S12] Taylor, J.M., Vaidhyanathan, R., Iremonger, S.S., and Shimizu, G.K. (2012). Enhancing water stability of metal-organic frameworks via phosphonate monoester linkers. J. Am. Chem. Soc. *134*, 14338-14340.

[S13] Yang, C., Kaipa, U., Mather, Q.Z., Wang, X., Nesterov, V., Venero, A.F., and Omary, M.A. (2011). Fluorous metal-organic frameworks with superior adsorption and hydrophobic properties toward oil spill cleanup and hydrocarbon storage. J. Am. Chem. Soc. *133*, 18094-18097.

[S14] Qian, X.K., Yadian, B.L., Wu, R.B., Long, Y., Zhou, K., Zhu, B., and Huang, Y.Z. (2013). Structure stability of metal-organic framework MIL-53 (Al) in aqueous solutions. Int. J. Hydrogen Energy *38*, 16710-16715.

[S15] Kang, I.J., Khan, N.A., Haque, E., and Jhung, S.H. (2011). Chemical and thermal stability of isotypic metal-organic frameworks: effect of metal ions. Chem. Eur. J. *17*, 6437-6442.

[S16] Mondloch, J.E., Katz, M.J., Planas, N., Semrouni, D., Gagliardi, L., Hupp, J.T., and Farha, O.K. (2014). Are Zr_6-based MOFs water stable? Linker hydrolysis *vs.* capillary-force-driven channel collapse. Chem. Commun. *50*, 8944-8946.

[S17] Reinsch, H., van der Veen, M.A., Gil, B., Marszalek, B., Verbiest, T., de Vos, D., and Stock, N. (2012). Structures, Sorption Characteristics, and Nonlinear Optical Properties of a New Series of Highly Stable Aluminum MOFs. Chem. Mater. *25*, 17-26.

[S18] Kim, S.N., Kim, J., Kim, H.Y., Cho, H.Y., and Ahn, W.S. (2013). Adsorption/catalytic properties of MIL-125 and NH_2-MIL-125. Catal. Today *204*, 85-93.

[S19] Furukawa, H., Gandara, F., Zhang, Y.B., Jiang, J., Queen, W.L., Hudson, M.R., and Yaghi, O.M. (2014). Water adsorption in porous metal-organic frameworks and related materials. J. Am. Chem. Soc. *136*, 4369-4381.

[S20] Han, S., Huang, Y., Watanabe, T., Dai, Y., Walton, K.S., Nair, S., Sholl, D.S., and Meredith, J.C. (2012). High-throughput screening of metal-organic frameworks for CO_2 separation. ACS Comb. Sci. *14*, 263-267.
